# The impact of oxygen content on *Staphylococcus epidermidis* pathogenesis in ocular infection based on clinical characteristics, transcriptome and metabolome analysis

**DOI:** 10.3389/fmicb.2024.1409597

**Published:** 2024-07-10

**Authors:** Hongling Lv, Wenjia Zhang, Zhu Zhao, Yingpu Wei, Zhengyilin Bao, Yizheng Li, Zhulin Hu, Deyao Deng, Wenli Yuan

**Affiliations:** ^1^Department of Clinical Laboratory, The Affiliated Hospital of Yunnan University, Kunming, China; ^2^Yunnan Eye Institute and Key Laboratory of Yunnan Province, Yunnan Eye Disease Clinical Medical Center, Affiliated Hospital of Yunnan University, Yunnan University, Kunming, China

**Keywords:** *S. epidermidis*, ocular infection, transcriptomic, metabolomics, oxygen content

## Abstract

**Introduction:**

This study aims to delineate the etiology and prevalence of isolated pathogens, along with the clinical characteristics of endophthalmitis patients over a 9-year period at hospital in Southwest of China. Additionally, we investigating the metabolic and cellular processes related to environmental factors may offer novel insights into endophthalmitis.

**Methods:**

We analyzed data pertaining to endophthalmitis patients treated at the Affiliated Hospital of Yunnan University from 2015 to 2023. According to our clinical data, we conducted an experiment based on transcriptomics and metabolomics analysis to verify whether environmental factors affect behavior of *S. epidermidis* by culturating *S. epidermidis* under oxic and microoxic condition.

**Results:**

In this study, 2,712 fungi or bacteria strains have been analyzed, gram-positive bacteria constituted 65.08%, with *S. epidermidis* being the most predominant species (25.55%). Ophthalmic trauma was the primary pathogenic factor for *S. epidermidis* ocular infections. Regarding fluoroquinolones, *S. epidermidis* exhibited the higher resistance rate to levofloxacin than moxifloxacin. Moreover, our investigation revealed that *S. epidermidis* in microoxic environment increase in energy metabolism, amino acid metabolism, and membrane transport.

**Conclusion:**

Our findings underscore the significance of *S. epidermidis* as a crucial pathogen responsible for infectious endophthalmitis. It is crucial to exercise vigilance when considering Levofloxacin as the first-line drug for empiric endophthalmitis treatment. The metabolites alteration observed during the commensal-to-pathogen conversion under microoxic condition serve as a pivotal environmental signal contributing to *S. epidermidis* metabolism remodeling, toward more pathogenic state.

## 1 Introduction

The severity of ophthalmic infections ranges widely from self-limiting bacterial conjunctivitis to potentially threatening conditions such as keratitis and endophthalmitis (Grandi et al., 2019). In recent years, the pathogen species that causes ocular infection is constantly changing along with antibiotic resistance rate (Hong, 2021), which will not only increase the difficulty of clinical diagnosis and treatment of diseases, but also prolong the recovery time of patients, thus affecting prognosis (Abebe et al., 2023). Hence, strict monitoring of ocular infection pathogens and antibiotic resistance combining with understanding the clinical characteristics of common pathogens may provide significant value to guide clinicians administrate early treatment for ocular infections. *Staphylococcus epidermidis* (*S. epidermidis*) is one of the most common isolates from ocular infections (Asadi-Amoli et al., 2022) since *S. epidermidis* is normal flora of human skin, including ophthalmic area (Polat et al., 2023). *S. epidermidis* mainly contributes to foreign body-associated infection, its attachment to the conjunctiva and skin around the eye makes it prone to contaminating medical equipment used in ocular surgery, leading to infections and inflammatory reactions within the eyes (Polat et al., 2023) but a few studies reveal it as infectious pathogen (Belyhun et al., 2018; Liu et al., 2020). Despite high prevalence rate of *S. epidermidis* in clinical specimens, whether the bacteria act as source of foreign objects or causative paothogen in ocular infection remains uncertain (Lin et al., 2021). For infectious agent perspective, identifying the molecular determinants and biomarkers for *S. epidermidis* transition from commensal to pathogen may offer potential therapeutic targets (du et al., 2021). According to the literature, the oxygen level in the posterior chamber is very low. In the anterior chamber, there is about a sevenfold oxygen gradient in the human cornea due to oxygen consumption by the corneal cells (Siegfried et al., 2010; Beebe et al., 2014). Therefore, the oxygen content altered when *S. epidermidis* enters eyes, due to shift from normoxic to microoxic environment. To understand the mechanisms of *S. epidermidis* adapt oxygen level changes during infection, we analyzed gene expression and metabolic pattern of *S. epidermidis* in microoxic and normoxic environments, respectively. The total RNA and metabolites have been extracted from *S. epidermidis* cultured under microoxic and normoxic environment respectively, to perform transcriptome and metabolome. The results indicated pathways and biological processes of bacteria rewired under low oxygen level leading to pathogenic transition from normal flora suggesting environmental oxygen concentration shift may be a key signal for pathogenicity of *S. epidermidis*.

## 2 Materials and methods

### 2.1 Retrospective analysis of clinical data

This study covered 2,708 ocular infection cases in the Affiliated Hospital of Yunnan University from 2015 to 2023. Data including patients’ medical history, underlying diseases, allergy history, pathogenic causes, pathogenic isolates, and results of antibiotic sensitivity tests have been collected. Ethical approval for this study was granted by Affiliated Hospital of Yunnan University hospital’s Ethics Committee. Bacterial identification and antibiotic sensitivity tests were conducted using VITEK 2 Compact automated microbial identification and drug sensitivity analysis system (Meyrié, French). The Clinical and Laboratory Standards Institute (CLSI) M100-S32 breakpoints were used for interpreting the results. Fungal identification has conducted according to colonic morphology and microstructure of mycelium and spores.

### 2.2 Bacteria growth

Ten strains of *S. epidermidis* were isolated from the eyes of patients with endophthalmitis for multilocus sequence typing (MLST), and a total of 5 STs were obtained (4 with ST2, 2 with ST23, 2 with ST9, 1 with ST59 and 1 with ST64) and lineage ST2 was the most frequent. According to this result, and combined with relevant literature reports on molecular and phenotypic characterization of *S. epidermidis* isolates from ocular infection (Flores-Páez et al., 2015), we selected the *S. epidermidis* with ST2 for the next experiment.

*S. epidermidis* were isolated from the vitreous of a patient with endophthalmitis in the Affiliated Hospital of Yunnan University. This strain was inoculated in Luria-Bertani (LB) liquid medium. For oxic conditions, LB broth containing *S. epidermidis* were incubated at 37°C in flask, with volume no exceeding up to 15%, and the oxygen source was ambient air, mainly including nitrogen 78% and oxygen 21%. For microoxic culture condition, the flask containing *S. epidermidis* and LB broth was placed in the MARK II AN2CTS anaerobic microaerobic bacteria culture system (MART Microbiology, Netherlands), and N_2_ was added to the culture system to reach 6% oxygen content, and then the culture system was incubated at 37°C. Bacteria then were harvested after culturing for 24 h, following centrifuge at 3,000 × g, 5 min. The supernatants were collected and into 1.5 mL tube, immediately froze in liquid nitrogen and stored at −80°C for metabolomics analysis. To perform transcriptomic analysis, liquid-cultured bacteria were subjected to centrifuge at 12,000 × g, 2 min, 4°C to harvest bacteria which froze in liquid nitrogen and store at −80°C.

### 2.3 Transcriptomic analysis

The total RNAs of oxic cultured group (z) and microoxic cultured group (w) were extracted using the Trizol Reagent (Thermo Fisher Scientific, United States) following the manufacturer instructions. The quality and quantity of RNA were measured using NanoDrop spectrophotometer (Thermo Fisher Scientific, United States) and Bioanalyzer 2100 system (Agilent, United States). Zymo-Seq RiboFree Total RNA Library Kit (Zymo, United States) was used to remove rRNA from total RNA. Sequencing was carried out on the NovaSeq 6000 platform (Shanghai Personalbio Technology Co., Ltd., China). Each treatment was carried out in triplicate.

Differential gene expression analysis was performed with the R package DESeq2 (Love et al., 2014). Wald test was used to identify genes that were differentially expressed between two *S. epidermidis* groups. Differentially expressed genes (DEGs) were identified based on adjusted *p* value < 0.05 and |log2 FoldChange| > 1 as selection criteria as described in a previous study (Wen et al., 2022). The cluster Profiler (version 4.2.2) package (Bioconductor, United States) was used to evaluate enrichment of the GO and KEGG in the sets of upregulated and downregulated genes separately (Wu et al., 2021). The categories with adjusted *p* value < 0.05 were considered as significantly enriched.

### 2.4 Untargeted metabolites analysis

Four hundred microliters of pre-cooled extraction reagent (methanol:acetonitrile:water = 2:2:1) were added in 100 μL supernatant of oxic and microoxic groups, after homogenizing for 5 min using Tissue Lyser (JXFSTPRP, China). Samples were sonicated for 10 min and incubate at −20°C for 1 h, then subjected to centrifuge at 3,000 × g for 15 min at 4°C, supernatants were transferred for vacuum freeze drying. The metabolites were re-suspended in 200 μL of 10% methanol and sonicated for 10 min at 4°C, followed with centrifuge for 15 min at 3,000 × g. The supernatants were transferred to auto-sampler vials for LC–MS analysis.

The samples were analyzed on a Waters 2D UPLC (Waters, United States) coupled to a Q-Exactive mass spectrometer (Thermo Fisher Scientific, United States) with a heated electrospray ionization (HESI) source and controlled by the Xcalibur 2.3 software program (Thermo Fisher Scientific, Waltham, MA, United States). Chromatographic separation was performed on a Waters ACQUITY UPLC BEH C18 column (1.7μm, 2.1 mm × 100 mm, Waters, United States) and the column temperature was maintained at 45°C.

The MS data processing was performed using The Compound Discoverer 3.1 (Thermo Fisher Scientific, United States) software, mainly included peak extraction, peak alignment, and compound identification. Data pre-processing, statistical analysis, metabolite classification annotations and functional annotations were performed using the self-developed metabolomics R package metaX and the metabolome bioinformatic analysis pipeline. The multivariate raw data was dimensionally reduced by PCA (Principal Component Analysis) to analyze the groupings, trends (intra- and inter-group similarities and differences) and outliers of the observed variables in the data set. Using PLS-DA (Partial Least Squares Method-Discriminant Analysis), the VIP (Variable Importance in Projection) values of the first two principal components of the model, combined with the variability analysis, the Fold change and the Student’s test to screen for differential metabolites. The metabolites with VIP > 1 and *p* < 0.05 (Student’s test) were considered as significantly changed metabolites.

### 2.5 Reverse-transcription quantitative polymerase chain reaction

We performed a qPCR assay to validate the gene expression changes obtained from the transcriptomic analysis. The total RNA of bacteria were extracted using the TsingZol total RNA Extraction reagent (Qingke, China), cDNA was synthesized using the Goldenstar^™^ RT6 cDNA Synthesis kit (Qingke, China), RT-qPCR was performed using 2 × T5 Fast qPCR Mix (Qingke, China) on ABI 7500 Real-Time PCR system (Thermo Fisher Scientific, United States). The primers used in this study were listed in Supplementary Table S1. The 2^−ΔΔCt^ approach was used to calculate the relative expression levels of differential genes among the groups. GraphPad Prism 7.0 and SPSS 21.0 were used to analyze data.

## 3 Results

### 3.1 Distribution of microorganisms in ocular infection

A total of 2,598 ocular infections cases were included in the study, of which 105 cases were co-infected with two species bacteria and 9 cases were co-infected with single specie bacteria and fungi. A total of 2,712 samples of microorganism were obtained. Bacteria constituted 81.08% (2,199 out of 2,712 isolates), subdivided into 1,765 Gram-positive and 434 Gram-negative isolates. Fungal isolates represented 18.92% (513 out of 2,712). Among these, the most prevalent Gram-positive bacteria was *S. epidermidis*, accounting for 25.55% (693 out of 2,712 isolates), whereas *Pseudomonas aeruginosa* was the predominant Gram-negative bacterium, constituting 2.25% (61 out of 2,712 isolates). The most common fungal isolate was *Fusarium* spp., representing 11.21% (304 out of 2,712 isolates), as detailed in Table 1.

**Table 1:** Distribution of microorganism in ocular infection (n/strain, %).

Microorganism	*N*	(%)
Gram-positive bacteria	1,765	65.08
*Staphylococcus epidermidis*	693	25.55
*Staphylococcus aureus*	139	5.13
Other coagulase negative staphylococci	246	9.07
*Enterococcus* spp.	65	2.40
*Streptococcus* spp.	378	13.94
Gram-positive bacilli	169	6.23
*Micrococcus luteus*	44	1.62
Actinomyces	5	0.18
Other Gram-positive cocci	9	0.33
Other Gram-positive bacilli	17	0.63
Gram-negative bacteria	434	16.00
*Escherichia coli*	41	1.51
Klebsiella	53	1.95
*Pseudomonas aeruginosa*	61	2.25
Acinetobacter	33	1.22
*Stenotrophomonas maltophilia*	23	0.85
Enterobacterium	54	1.99
*Serratia* spp.	26	0.96
Other Gram-negative bacilli	128	4.72
Other Gram-negative cocci	15	0.55
Fungus	513	18.92
*Candida* spp.	69	2.54
*Fusarium* spp.	304	11.21
*Aspergillus* spp.	74	2.73
Other filamentous fungi	66	2.43
Total isolated pathogens	2,712	100.00

### 3.2 Specimen types of ocular infection

In this study, of the 2,712 microorganism samples were isolated from ocular infections, 2,117 (78.06%) were derived from corneal and conjunctival secretions, 555 (20.46%) from vitreous humor, and 40 (1.47%) from aqueous humor and ocular tissues. Notably, *S. epidermidis*, the most prevalent bacteria in ocular infections, were predominantly isolated from corneal and conjunctival secretions, comprising 71.14% (493 out of 693 isolates).

### 3.3 Retrospective analysis of contagious risk factors in ocular infection

The transmission route of *S. epidermidis*, *Fusarium* spp., and *P. aeruginosa* in ocular infections were reviewed. Among collected ocular infection cases, patients who were infected with any of *S. epidermidis*, *Fusarium* spp., and *P. aeruginosa* with a history of ophthalmic trauma, predominantly male, accounted for 48.21% of all cases. Additionally, infections were associated with different medical histories have revealed that 20.42% had keratitis or corneal ulcers, 12.72% had dacryocystitis or dacryocyst obstruction, and 10.38% had undergone intraocular surgery, including 6.81% with a history of cataract surgery. These findings are detailed in Table 2. Among the identified infectious factors, ocular trauma was the predominant contributor to *S. epidermidis* infections, accounting for 51.53%. Based on clinical diagnoses and treatment outcomes, certain bacteria were classified as commensal micro-organisms and thus excluded from the statistical analysis.

**Table 2:** Pathogenic factors of common ocular infections.

Pathogenic factor	*S. epidermidis*		Fusarium spp.		*P. aeruginosa*		Total	
	*N*	(%)	*N*	(%)	*N*	(%)	*N*	(%)
Ocular trauma (total)	320	51.53	88	38.60	24	51.06	432	48.21
Penetrating wound and contused	291	46.86	51	22.37	15	31.91	357	39.84
Intraocular foreign bodies	24	3.86	35	15.35	8	17.02	67	7.48
Chemical injury	5	0.81	2	0.88	1	2.13	8	0.89
Postoperative (total)	74	11.92	13	5.70	6	12.77	93	10.38
Post-cataract surgery	51	8.21	8	3.51	2	4.26	61	6.81
Post-prosthetic eye implantation	4	0.64	0	0.00	2	4.26	6	0.67
Post-vitrectomy	2	0.32	0	0.00	1	2.13	3	0.33
Post-keratoplasty	4	0.64	3	1.32	0	0.00	7	0.78
After other eye surgery	13	2.09	2	0.88	1	2.13	16	1.79
Orbital cellulitis-related	24	3.86	1	0.44	2	4.26	27	3.01
Dacryocystitis/dacryocyst obstruction	112	18.04	1	0.44	1	2.13	114	12.72
Keratitis/corneal ulcer	51	8.21	122	53.51	10	21.28	183	20.42
Other causes	40	6.44	3	1.32	4	8.51	47	5.25
Total	621	100.00	228	100.00	47	100.00	896	100.00

Due to the difficulty in follow-up of some patients, some clinical data were missing.

Correlation analysis of infectious factors in ocular infections showed that *Fusarium spp*-related ocular infection mainly occurred in cases involving keratitis/corneal ulcers (*r*_s_ = 0.479, *p* < 0.001).

### 3.4 Clinical characteristics of ocular infections

The clinical characteristics of patients with ocular infections caused by *S. epidermidis*, *Fusarium* spp., and *P. aeruginosa* were analyzed using chi-square tests. Comparative analysis revealed that infections caused by *Fusarium* spp. were more prevalent in female patients compared to those caused by the other two pathogens. The higher proportion of patients aged 60 years or older were affected by *P. aeruginosa*-related ocular infections. Non-farmers with a history of smoking and drinking were more commonly infected with *S. epidermidis*. Post-ocular surgery infections were predominantly associated with *S. epidermidis* and *P. aeruginosa*. Regarding the duration of infection, *P. aeruginosa* infections typically had a shorter course, whereas *Fusarium* spp. infections tended to be longer. Most *S. epidermidis* infections (47.8%) lasted ≤ 1 week, yet a significant portion of overall infections (44.3%) persisted for ≥ 2 weeks, as detailed in Table 3.

**Table 3:** Clinical characteristics of patients with ocular infection caused by different pathogens.

Factor (*n*, %)		*S. epidermidis*	Fusarium spp.	*P. aeruginosa*	χ^2^	*p*
		(*n* = 621)	(*n* = 228)	(*n* = 47)		
Gender	Male	408 (65.7)	129 (56.6)	36 (76.6)	0.945	0.009
	Female	213 (34.3)	99 (43.4)	11 (23.4)		
Age	≥60y	138 (22.2)	58 (25.4)	21 (44.7)	12.256	0.002
	<60y	483 (77.8)	170 (74.6)	26 (55.3)		
Profession	Farmer	259 (41.7)	158 (69.3)	30 (63.8)	54.640	<0.001
	Other	362 (58.3)	70 (30.7)	17 (36.2)		
Duration of disease	≤1 week	297 (47.8)	16 (7.0)	26 (55.3)	154.272	<0.001
	1–2 weeks	49 (7.9)	64 (28.1)	12 (25.5)		
	≥2 weeks	275 (44.3)	148 (64.9)	9 (19.1)		
Underlying disease	Hypertension	61 (9.8)	22 (9.6)	8 (17.0)	2.567	0.277
	Diabetes	27 (4.3)	7 (3.1)	4 (8.5)	2.897	0.235
	Diseases of immune system	4 (0.6)	2 (0.9)	1 (2.1)	0.933	0.627
Personal history	Smoking history	200 (32.2)	53 (23.2)	14 (29.8)	6.401	0.041
	Drinking history	139 (22.4)	27 (11.8)	6 (12.8)	13.269	0.001
	Allergic history	59 (9.5)	20 (8.8)	4 (8.5)	0.140	0.932
History of surgery	Ocular surgery history	145 (23.3)	24 (10.5)	14 (29.8)	19.548	<0.001

### 3.5 Drug resistance analysis of ocular infection associated *Staphylococcus epidermidis*

The antibiotic sensitivities results of *S. epidermidis* were summarized in Table 4. Of the 468 isolates of *S. epidermidis* analyzed (some lacking drug sensitivity results), resistance rates to vancomycin, linezolid, tigacycline, furantoin, and quinupristin/dalfopristin were all below 3.0%. Resistance rates for gentamicin and rifampicin were below 10.0%, while those for erythromycin and penicillin were notably higher, >50.0% and >80.0%, respectively. The Chi-square trend tests for annual trends in antimicrobial resistance found that the resistance rates of *S. epidermidis* to levofloxacin (*p* = 0.001) and ciprofloxacin (*p* = 0.002) showed an increasing trend.

**Table 4:** Antimicrobial resistance of ocular *S. epidermidis in* 2015–2023 (%).

Antibiotics	2015	2016	2017	2018	2019	2020	2021	2022	2023
	(*n* = 19)	(*n* = 58)	(*n* = 43)	(*n* = 55)	(*n* = 66)	(*n* = 37)	(*n* = 62)	(*n* = 73)	(*n* = 55)
Benzoxicillin	73.6	67.2	65.1	65.4	48.4	64.8	62.9	71.2	80.0
Cefoxitin	26.4	32.8	34.9	34.6	51.6	31.5	36.1	28.8	20.0
Clindamycin	68.4	51.7	51.2	50.0	39.3	37.8	43.5	49.3	49.0
Gentamicin	9.0	9.8	6.9	7.2	7.5	0.0	4.8	0.0	5.4
Moxifloxacin	15.7	12.0	6.9	18.1	16.6	19.6	14.5	17.8	18.1
Levofloxacin	52.6	48.2	41.8	47.2	30.3	48.6	59.6	67.2	63.6
Ciprofloxacin	31.5	36.2	30.2	41.8	28.7	37.8	48.3	54.7	49.0
Penicillin	100.0	94.8	83.7	90.9	87.8	94.4	93.5	97.2	96.3
Rifampin	5.2	1.7	4.6	1.8	1.5	0.0	1.6	6.8	9.9
Vancomycin	0.0	0.0	2.3	0.0	0.0	2.7	0.0	0.0	0.0
Erythromycin	89.4	74.1	74.4	69.0	66.6	54.0	59.6	80.8	65.4
Linezolid	0.0	0.0	0.0	0.0	0.0	0.0	0.0	0.0	0.0
Trimethoprim/sulfa	63.1	62.0	51.1	61.8	39.3	48.6	67.7	52.0	60.0
Tetracycline	52.6	55.1	53.4	47.2	46.9	51.3	46.7	53.4	56.3
Tigacycline	0.0	0.0	0.0	0.0	0.0	0.0	0.0	0.0	0.0
Furantoin	0.0	0.0	2.3	0.0	0.0	0.0	0.0	0.0	0.0
Quinupristin/Dalfopristin	0.0	0.0	2.3	0.0	0.0	0.0	1.6	1.3	1.8

### 3.6 Transcriptomic analysis of ocular infection associated *Staphylococcus epidermidis*

**Figure 1:**
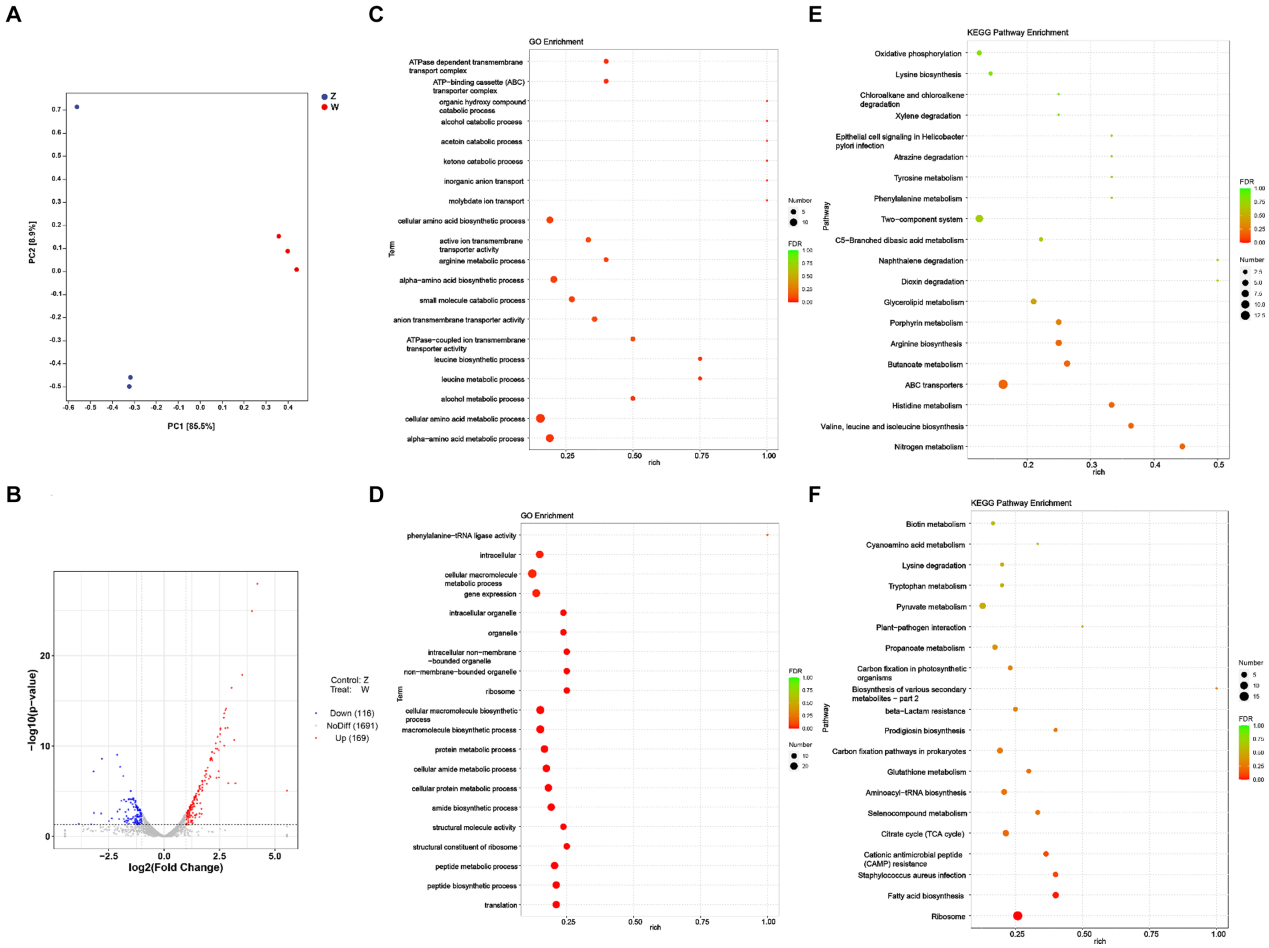
Transcriptomic analysis of *S. epidermidis* in oxic (z) versus microoxic group (w). **(A)** Principal-component analysis (PCA) of the DEGs in two compared groups. The closer the distances represent higher the similarity between the groups. Each scatter plot point represents a sample, with shape and color denoting different experimental groups. **(B)** Volcano plot of differentially expressed genes of *S. epidermidis* in two compared groups. Displayed two vertical dotted lines represent a two-fold expression difference threshold, with a dotted line indicating a *p* value threshold of 0.05. Red dots represent up-regulated genes, blue dots down-regulated genes, and gray dots non-significantly differentially expressed genes. **(C)** Gene Ontology (GO) classification of all of the up-regulated DEGs in two compared groups. **(D)** GO classification of all of the down-regulated DEGs in two compared groups. **(E)** The top 20 enriched KEGG pathway analysis of all of the up-regulated DEGs in two compared groups. **(F)** The top 20 enriched KEGG pathway analysis of all of the down-regulated DEGs in two groups.

Principal Component Analysis (PCA) delineated transcriptomic differences between the oxic (z) and microoxic (w) groups and variance within replicate samples (Figure 1A), one sample from Z does not group with the other samples of that condition in Figure 1A, PCA reflects the repeatability of the sample, and this project does have a set of sample PCA compared with the other two discrete points, which may be relatively this sample discrete points. However, in this project, the two groups were basically separated, and when the differences were screened, the difference analysis software would also consider the intra-group repeats and inter-group differences to analyze fc and *p* values. In addition, we had the verification of qPCR, which should be able to say the reliability of the sequencing results. Transcriptomic analysis identified 285 differentially expressed genes (DEGs) involved in respiratory and energy metabolism, cell wall biosynthesis, among others. One hundred and sixty-nine genes were significantly up-regulated and 116 genes down-regulated based on |log2FoldChange| > 1 and *p* value < 0.05 (Figure 1B; Supplementary Table S2). Further transcriptome data analysis included Gene Ontology (GO) and Kyoto Encyclopedia of Genes and Genomes (KEGG) enrichment analyses. The GO analysis encompassed biological process (BP), cellular component (CC), and molecular function (MF) categories, revealing significant enrichment 35 genes in BP, 2 in CC, and 13 in MF categories (*p* value < 0.05). The top 20 enriched GO pathways were illustrated in a bubble chart with the lowest false discovery rate (FDR) values (Figures 1C,D). Similarly, the top 20 KEGG pathways, displaying the most significant enrichment, were depicted in a bubble chart (Figures 1E,F). GO analysis showed up-regulated DEGs were particularly enriched in processes like alpha-amino acid metabolism, cellular amino acid metabolism, alcohol metabolism, leucine metabolism, ATPase-coupled ion transmembrane transporter activity, down-regulated DEGs were notably enriched in translation, peptide biosynthesis, ribosome structure, and amide biosynthesis. KEGG analysis revealed upregulation in nitrogen metabolism, valine, leucine and isoleucine biosynthesis, histidine metabolism, ABC transporters, butanoate metabolism, and arginine biosynthesis. In contrast, down-regulated DEGs were primarily associated with ribosome and fatty acid biosynthesis pathways.

### 3.7 Metabolomic analysis of ocular infection associated *Staphylococcus epidermidis*

**Figure 2:**
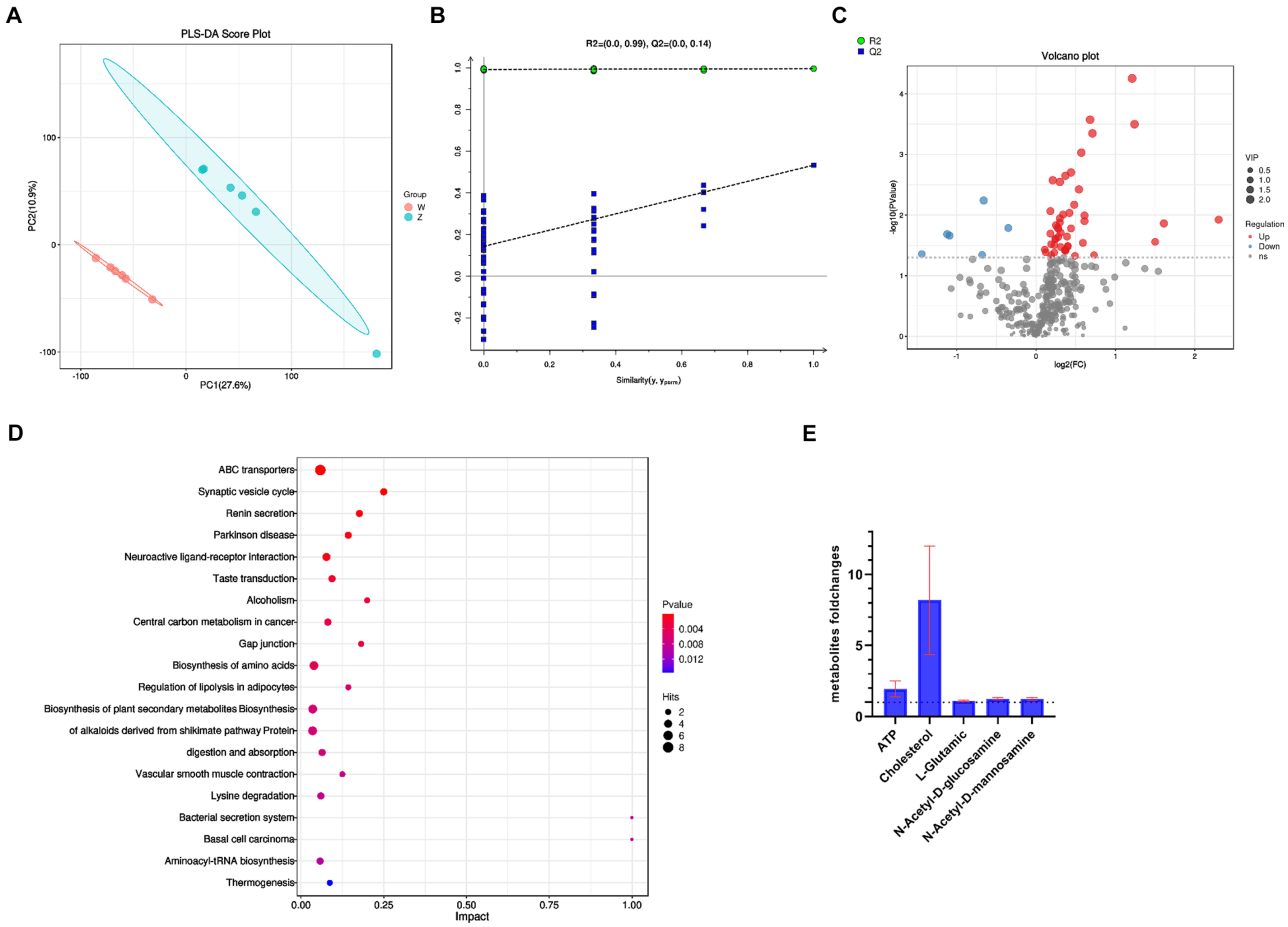
Metabolomics of *S. epidermidis* in oxic (z) and microoxic group (w). **(A)** PLS-DA score plot in negative ion scanning mode. Horizontal axis indicates the predicted principal component scores of the first principal component, demonstrating between-sample group differences. Vertical axis indicates the second principal component scores, demonstrating within-sample group differences, with each scatter plot representing a sample, shape and color indicating different experimental groups. **(B)** Permutation test of OPLS-DA in negative ion scanning mode. The results are reliable and valid if any of the following points are satisfied: (1) All blue Q2 points are lower than the rightmost blue Q2 point. (2) The regression line of a point crosses the ordinate or is less than 0. **(C)** The volcanic map presents differential metabolites of *S. epidermidis* in two comparative groups. Each dot symbolizes a distinct metabolite. Metabolites significantly up-regulated are marked in red, while those significantly down-regulated are in blue. Metabolites without significant differences are represented in gray, determined by a variable importance in projection (VIP) greater than 1 and a *p* value less than 0.05. **(D)** The top 20 enriched KEGG pathways sorted by *p* value of the two compared groups. The size of point indicates the number of enriched metabolites. **(E)** Fold changes (w/z) of differential metabolites of oxic (z) versus microoxic group (w), ATP, cholesterol, L-glutamic acid, N-Acetyl-D-glucosamine and N-Acetyl-D-mannosamine were all increased in the microoxia group of *S. epidermidis.*

Partial Least Squares Discriminant Analysis (PLS-DA) was used to model the relationship between metabolite production and sample categories (Figure 2A). The model’s robustness was supported by Variable Importance in Projection (VIP) scores, aiding in the identification of differential metabolites. This model exhibited strong explanatory and predictive capabilities, with R2Y = 0.995 and Q2Y = 0.792. Typically, R2 and Q2 values above 0.5 are considered favorable, and a minimal difference between them is desired. The Orthogonal Partial Least Squares Discriminant Analysis (OPLS-DA) results in the negative ion mode (NEG) demonstrated clear differentiation between the two sample groups, as confirmed by permutation tests (Figure 2B). Metabolomics analysis revealed 52 DEMs in the microoxic group (w) compared to the oxic group (z). Among these, 46 DEMs were significantly up-regulated and 6 down-regulated, based on VIP > 1 and *p* < 0.05 (Supplementary Table S3). Minor variances in metabolic characteristics of *S. epidermidis* under oxic and microoxic conditions were observed (Figure 2C). Further investigation into the metabolic pathways involving DEMs was conducted through KEGG pathway enrichment analysis, identifying 35 enriched pathways, including ABC transporters, synaptic vesicle cycle, and renin secretion (Figure 2D). These findings suggest that environmental information processing plays a significant role in the microoxic metabolism of *S. epidermidis.*

### 3.8 Integrative analysis of metabonomics and transcriptomics

**Figure 3:**
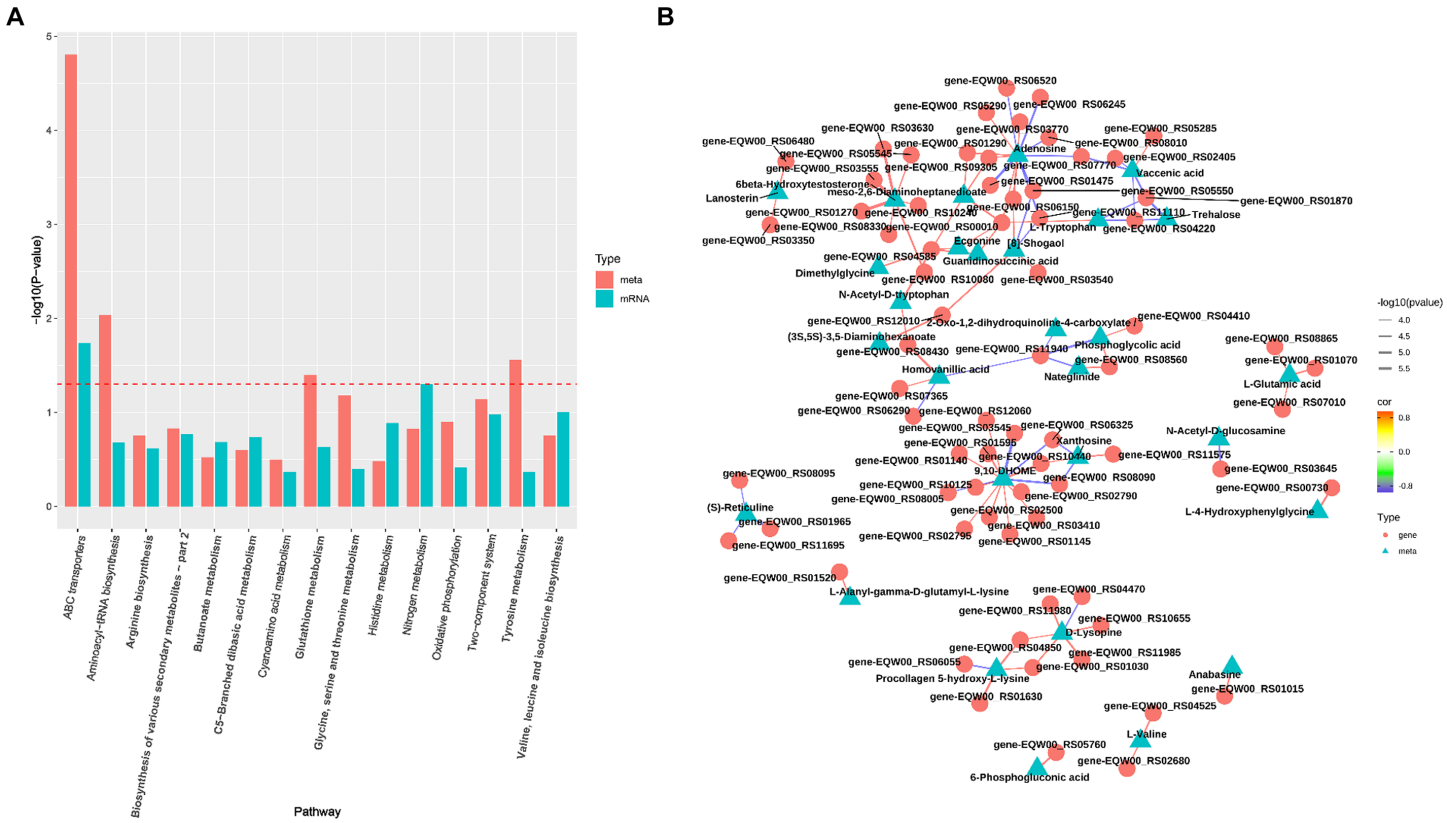
Integration analysis of transcriptomics and metabolomics *S. epidermidis* in oxic (z) and microoxic group (w). **(A)** The analysis identified pathways enriched with both genes and metabolites. The horizontal axis represents the names of metabolic pathways, while the vertical axis denotes the *p* value from the dual-omics enrichment analysis. Different omics are distinguished by varying colors, and the red dotted line marks the *p* = 0.05 threshold. Results positioned above this line indicate a *p* value of less than 0.05. **(B)** The top100 correlation network graph with the smallest *p* value, the color of the line represents the size of the correlation coefficient, and the thickness represents the size of the *p* value, the triangular nodes represent metabolites, and the circle nodes represent transcripts.

To elucidate the relationship between transcriptomic and metabolomic alterations in *S. epidermidis* under microoxic conditions, we conducted an integrative analysis using MetaboAnalyst Joint-Pathway analysis. This analysis aimed to identify molecular pathways implicated in metabolic dysregulation within the microoxic environment. Our findings revealed 39 enriched pathways in both the transcriptome and metabolome of the microoxic group (Figure 3A). Specifically, six pathways, ABC transporters, aminoacyl-tRNA biosynthesis, purine metabolism, amino sugar and nucleotide sugar metabolism, lysine degradation, and central carbon metabolism exhibited significant alterations. These changes were determined based on criteria of DEGs number > 3, DEMs number > 3 and FDR < 0.01. Additionally, correlation networks illustrating interactions between genes and metabolites in these common pathways, with correlation coefficients exceeding 0.8, were constructed (Figure 3B). This shows that microoxic environment modulates gene expression and metabolite features of *S. epidermidis*, which were linked to membrane transport, translation, nucleotide metabolism, carbohydrate metabolism and amino acid metabolism.

### 3.9 The validation of the gene expression changes obtained from the transcriptomic analysis

**Figure 4:**
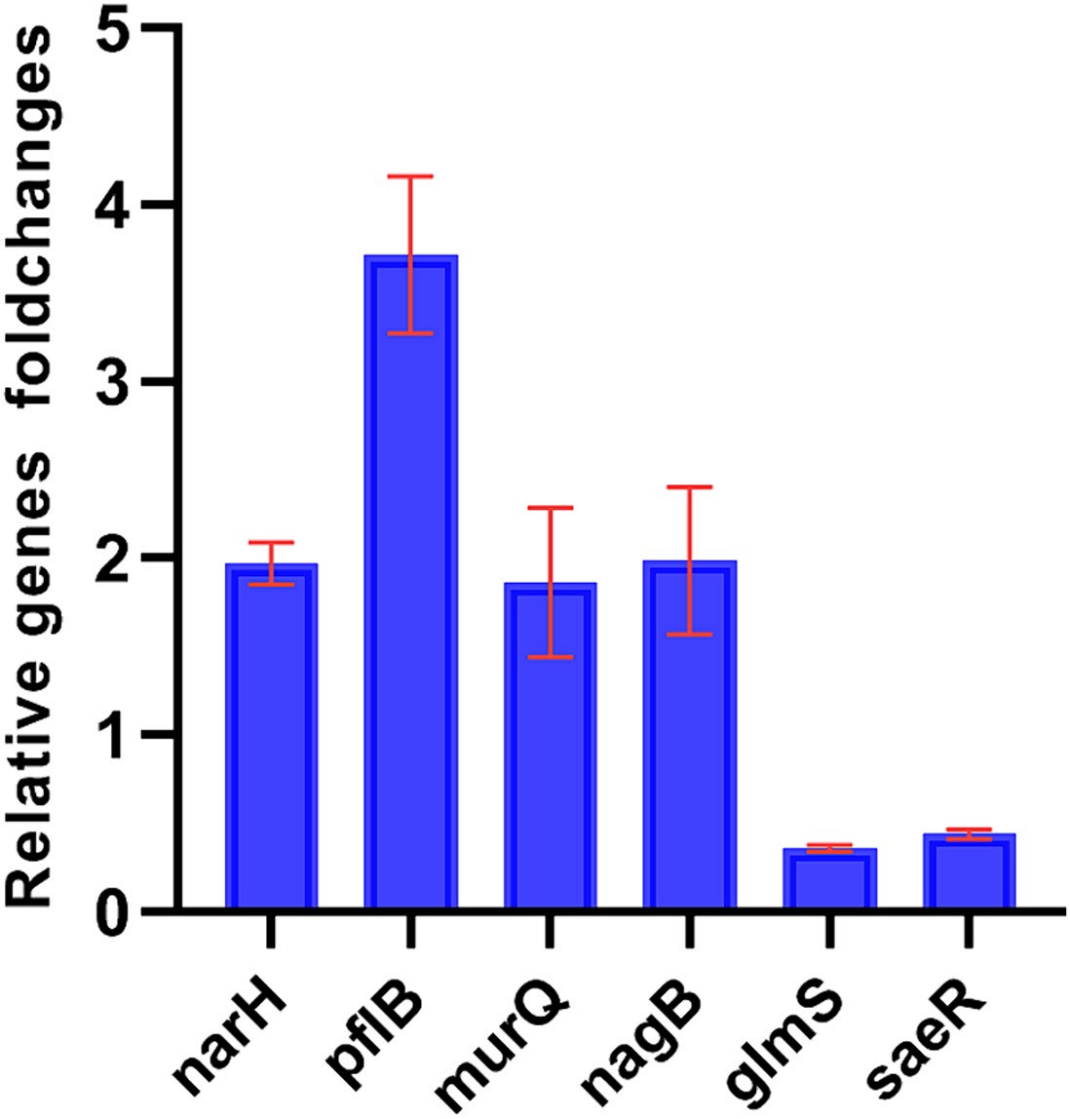
Fold changes (w/z) of DEGs of oxic (z) versus microoxic group (w). Fold changes (w/z) of differential genes expression of oxic (z) versus microoxic group (w). narH, pflB, murQ, nagB genes were up-regulated in microoxic group of *S. epidermidis*, while glmS, saeR genes were down-regulated in microoxic group.

Six genes were selected to verify the accuracy of the transcriptome data by qPCR. While the fold change in genes of the two data sets was different, the results of qPCR showed that the genes trended consistently with the transcriptome data. The result showed that the expression of narH, pflB, murQ, nagB were upregulated and glmS, saeR were downregulated, confirming the validity of the transcriptome data (Figure 4).

## 4 Discussion

This comprehensive study analyzes bacterial and fungal profiles of ocular infections, representing a significant contribution to existing literature. The most frequently isolated Gram-positive bacterium was *S. epidermidis* (25.55%), while *P. aeruginosa* (2.25%) and *Fusarium* spp. (11.21%) were the predominant Gram-negative bacterium and fungus, respectively. Notably, *S. epidermidis* was the most commonly detected microorganism in ocular infections, consistent with prior study (Shams Abadi et al., 2023). The majority of ocular infection specimens (78.06%) originated from corneal and conjunctival secretions, primarily due to the ease of sample collection.

In a retrospective analysis of clinical data from patients with ocular infections, we examined 621 isolates of *S. epidermidis*, 228 isolates of *Fusarium* spp., and 47 isolates of *P. aeruginosa*. Approximately half of the ocular infection cases (48.21%) were attributable to open trauma, predominantly among male patients. This aligns with Qing L et al.’s findings that posttraumatic endophthalmitis accounted for 49.62% of infectious endophthalmitis cases at the Zhongshan Ophthalmic Center (Liu M. et al., 2022; Liu Q. et al., 2022). Ocular surgery is a recognized high-risk factor for ocular infection (Reddy et al., 2015). In our study, *S. epidermidis* and *P. aeruginosa* infections occurred frequently during post-surgery, corroborating with other reports of *S. epidermidis* as a common post-operative endophthalmitis causative agent (Yannuzzi et al., 2017). In an Argentinian tertiary hospital, *P. aeruginosa* was identified as the primary microorganism in post-cataract surgery endophthalmitis cases (45.4%) (Segretín Gutiérrez et al., 2022). Moreover, *Fusarium* spp. and *P. aeruginosa* infections were prevalent among farmers, supporting the association between agricultural work and ocular infection (Toh et al., 2022). Interestingly, about half of *S. epidermidis* ocular infections persisted for more than 2 weeks, indicating their severity.

The global rise in antibiotic resistance is alarming, partially attributed to excessive and inappropriate antibiotic use (Ghweil et al., 2022; Mitra et al., 2022). In this study, *S. epidermidis* exhibited high sensitivity to tigacycline, linezolid, vancomycin, quinupristin/dalfopristin, rifampicin, furantoin, and gentamicin. However, clindamycin resistances were > 37.0% annually, surpassing the 25% rate reported elsewhere (Laura et al., 2020). Fluoroquinolones were widely used for the treatment and prevention of ocular bacterial infection, in this study, the resistance rates of *S. epidermidis* to levofloxacin were >30.0%, and to moxifloxacin were <20.0%, suggesting a decline in levofloxacin’s efficacy as a first-line treatment for empiric endophthalmitis. The cause of the high resistance of *S. epidermidis* strains to levofloxacin is probably related to the increased use of topical antibiotics, that is, the use of levofloxacin as an ophthalmic antibiotic. It is known that the prescription dose and resistance to antibiotics are closely related (Zeng et al., 2019). As for whether there was horizontal transmission within hospitals of resistant *S. epidermidis*, further study is needed. In the meantime, the rising trend of the resistance rates of *S. epidermidis* to levofloxacin and ciprofloxacin suggest that ophthalmologists should pay attention to the changing trends of pathogen distribution and their drug resistance patterns and choose sensitive antibiotics based on the local data.

It is essential to consider whether *S. epidermidis* isolated from the healthy conjunctiva is causative agent in infection (Kang et al., 2020). Most clinical studies have reported less severe endophthalmitis caused by *S. epidermidis* compared to *S. aureus* (Spoto et al., 2022). However, in our study, nearly half of the patients with *S. epidermidis* ocular infections experienced prolonged symptoms (over 2 weeks), including ocular pain and deteriorating vision. Understanding how *S. epidermidis* adapts to environmental changes during infection onset and its transition from commensal to pathogen, remains elusive. One significant environmental change is that shift in oxygen concentration from the ocular surface to the intraocular. Our hypothesis posited that microoxic conditions alter *S. epidermidis* gene expression, transcription, and metabolism, facilitating bacterial pathogenic conversion. To verified this, we analyzed differential transcriptomics and metabolomics under oxic and microoxic conditions, revealing the bacterial responses to environmental oxygen concentration shifts akin to those experienced during intraocular invasion. Bacteria transfer from the ocular surface to the intraocular is faced with changes in many environmental factors, such as pH, oxygen concentration, immune response, availability of nutrients, etc. (Ersan et al., 2015; Agujetas et al., 2018; Braunger et al., 2023), and one change in oxygen concentration is just one of the environmental factors, this condition is not fully representative of the complex ocular surface and intraocular environment. Recent studies also suggest that microaerobic lifestyles are important in host-associated microbial communities and the prevalence of microoxygen environments (Morris and Schmidt, 2013; Pedroza-Dávila et al., 2020), so we selected changes in environmental oxygen concentration as the experimental variable.

*S. epidermidis*, facultative anaerobic bacteria, possesses a versatile respiratory enzyme system for aerobic oxidation and anaerobic glycolysis. Environmental changes significantly impact microbial biological function (Burian et al., 2022). GO term analysis showed increased anaerobic respiration and decreased aerobic respiration in microoxic conditions. Genes related to anaerobic respiration (narH, pflB) were up-regulated, suggesting an adaptive response for better survival in microoxic environments. Moreover, ATP accumulation and up-regulation of the purine metabolic pathway were observed, indicating higher cellular energy availability in microoxic conditions.

Transcriptome and metabolome analysis revealed that *S. epidermidis* in a microoxic condition experiences altered gene expression related to environmental alteration. KEGG analysis showed up-regulation gene expression in amino acid metabolism and transmembrane transporter activity, suggesting increase in amino acid and energy metabolism, membrane transport, and metabolism of cofactors and vitamins under oxygen limitation. Additionally, regulatory systems for virulence in *S. epidermidis* were enhanced compared to oxic conditions. Notably, PhoR and SaeR of the PhoPR and SaeRS Two-Component Systems, which regulate a wide array of cell surface and secreted virulence factors, were up-regulated. PhoPR is involved in phosphate transport under phosphate-limiting conditions, responding to wall teichoic acid metabolism (Devine, 2018). The SaeRS system in *S. epidermidis* was found to negatively regulate genes involved in competence (comF, murF), cytolysis (lrgA), and autolysis (lytS) (Lou et al., 2016). Additionally, lytS belonging to the LytTR family, involved in cell autolysis regulation, increased in microoxic conditions. The expression of FsrB/D and SprE in *S. epidermidis*, crucial for gelatinase activity, serine protease production and biofilm formation, was also enhanced (Hashem et al., 2021). AgrB, associated with virulence and biofilm formation, was upregulated under microoxic conditions (Grande et al., 2014). Our findings suggest that *S. epidermidis* might shift from commensal to pathogen by enhancing nutrient metabolism and increasing virulence genes expression.

Metabolomic and transcriptomic analyses revealed that the valine, leucine, and isoleucine biosynthesis pathway was up-regulated in microoxic models. Branched-chain amino acids (BCAAs) are implicated in numerous physiological processes, including inflammation regulation. Elevated BCAA levels may activate the NF-κB signaling pathway and inflammasome (Ye et al., 2020). In microoxic environments, we estimated L-glutamic acid accumulation, since genes expression related to alanine, aspartate, and glutamate metabolism were up-regulated. L-glutamic acid damage retinal ganglion cells, inhibit axonal growth, elevate inflammatory and glial-cell related genes expression have been reported previously (Liu M. et al., 2022; Liu Q. et al., 2022). ATP and glutamate induce proinflammatory factor release from microglia, including TNF (tumor necrosis factor), IL-1β (interleukin 1 beta), and NO (nitric oxide) (Jesudasan et al., 2021). Moreover, N-Acetyl-D-glucosamine (GlcNAc) and N-Acetyl-D-mannosamine (ManNAc) may accumulate as result of up-regulated murQ, nagB and down-regulated glmS in the amino and nucleotide sugar metabolism pathway. ManNAc and GlcNAc are precursors of N-acetylneuraminic acid (NeuAc), a determinant of bacterial pathogenicity (Revilla-Nuin et al., 2002). These metabolic changes (Figure 2E) under microoxic conditions may contribute to *S. epidermidis* pathogenic transformation.

In the eye, the innate immune response is triggered by pathogen-associated molecular patterns (PAMPs) of bacteria, fungi, and viruses. Bacterial cell wall components like lipopolysaccharide, peptidoglycan, and teichoic acid activate pattern recognition receptors (PRR), such as Toll-like receptors (TLRs) to initiate signaling cascades that lead to chemokine and cytokine expression (Xia et al., 2021). Various ocular cells, including retinal pigment epithelial (RPE) cells, astrocytes, corneal epithelium, iris epithelium, retinal microglia, and Muller cells, express TLRs (Miller et al., 2019). The clearance of bacteria may cause host-mediated ocular damage when the inflammatory response is vigorous. Metabolites of *S. epidermidis* in microoxic conditions may also play a significant role in ocular infection-induced cytokine storms. Notably, an increase in cholesterol levels was observed under microoxic conditions. Increased cholesterol amplifies TLR signaling, thereby increasing cytokine and chemokine production and intensifying the inflammatory process (Tabas and Bornfeldt, 2016). Cholesterol also stimulate formation of NLRP3 inflammasome and IL-1β production (Chiu et al., 2021), induce inflammatory cytokine expression in human retinal pigment epithelium cells via the NF-κB pathway (Hu et al., 2014). Therefore, current bacterial endophthalmitis treatment, involving intravitreal antibiotic injections, fails to address inflammation-mediated ocular tissue damage, resulting in partial or complete vision loss (Das et al., 2021, 2023).

Emerging evidences suggest that repolarizing immune cells toward a less inflamed phenotype by manipulating metabolism with small molecules and intermediates (Reithuber et al., 2021). In our study, we noted an increase in glycolysis and gluconeogenesis under microoxic conditions. In cases of bacterial endophthalmitis, both resident and infiltrating cells show heightened glycolysis, interestingly, inhibiting glycolysis in this condition reduces intraocular inflammation (Francis et al., 2020), suggesting a potential therapeutic target for ocular infections. Hyperbaric oxygen therapy, used either as a primary or adjunctive treatment, addresses a range of medical disorders, including ocular conditions (Worley et al., 2020). Additionally, the suppression of the intraocular inflammatory response, which can cause secondary intraocular damage, through the use of intravitreal steroids may consider as complement antibiotic treatment (Jamil et al., 2023).

Therefore, we concluded that in *S. epidermidis*-related ocular infections, specific metabolites and up-regulated virulence genes may contribute to the bacterial transition from commensal to pathogenic state. However, this study is subject to several limitations. Firstly, the potential for erroneous records or missing data in medical records cannot be disregarded. Secondly, the research was conducted in a single center without including other areas of Yunnan province, the findings might not fully encapsulate the epidemiological profile of ocular infections across China.

## 5 Conclusion

In summary, we have isolated 2,712 sample of microorganisms from 2,598 patients with ocular infections with *S. epidermidis* constituting 25.55%. *S. epidermidis* ocular infections were predominantly found in men under 60 years of age and non-farmers, which correlate with a history of ocular operations.

Furthermore, our study discovered that the microoxic environment alters the gene expression and metabolism of *S. epidermidis*, facilitating its transition from commensal to pathogen. This finding underscores the potential of immune metabolism as new therapeutic target for endophthalmitis. We have investigated the risk factors of patients with ocular infections and metabolomics and transcriptomics of *S. epidermidis*. However, these culture conditions are not representative of the complex ocular surface and intraocular environment. Therefore, some of the pathways and functions altered in our experiments might be tested under specific growth conditions. In addition, the interaction of *S. epidermidis* microoxic metabolites with host epithelial cells remains unclear. Further research is required to unravel how bacterial metabolites impact on transition from commsal to pathogenic bacteria as well as microbe-host interactions.

## Data availability statement

Publicly available datasets were analyzed in this study. This data can be found here: The raw data from the transcriptomic experiment have been deposited in the SRA database of NCBI under SRA accession no. PRJNA956543 (https://www.ncbi.nlm. nih.gov/guide/). The raw data from the metabolomic experiment have been deposited into CNGB Sequence Archive (CNSA) of China National GeneBank DataBase (CNGBdb), the accession number of *S. epidermidis* under oxic and microoxic was CNP0004301.

## Ethics statement

The studies involving humans were approved by the affiliated hospital of Yunnan University Biomedical Ethics Committee. The studies were conducted in accordance with the local legislation and institutional requirements. The participants provided their written informed consent to participate in this study. Written informed consent was obtained from the individual(s) for the publication of any potentially identifiable images or data included in this article.

## Author contributions

HL: Conceptualization, Data curation, Project administration, Supervision, Validation, Writing – original draft. WZ: Conceptualization, Investigation, Project administration, Software, Supervision, Writing – review & editing. ZZ: Formal analysis, Investigation, Project administration, Validation, Writing – review & editing. YW: Data curation, Formal analysis, Project administration, Supervision, Validation, Writing – review & editing. ZB: Data curation, Formal analysis, Project administration, Validation, Writing – review & editing. YL: Data curation, Formal analysis, Methodology, Validation, Writing – review & editing. ZH: Formal analysis, Funding acquisition, Resources, Visualization, Writing – review & editing. DD: Funding acquisition, Project administration, Resources, Supervision, Visualization, Writing – review & editing. WY: Funding acquisition, Methodology, Project administration, Resources, Validation, Visualization, Writing – review & editing.
