# Supplementary material for: The impact of oxygen content on Staphylococcus epidermidis pathogenesis in ocular infection based on clinical characteristics, transcriptome and metabolome analysis
Source: Front Microbiol. 2024 Jul 10;15:1409597. doi: 10.3389/fmicb.2024.1409597 (PMC11266177; doi:10.3389/fmicb.2024.1409597)
Supplement: Supplementary file 1 [file Data_Sheet_1.pdf]

Table S1 Forward and reverse primer sequences for quantitative real-time polymerase chain reaction (qPCR) .

Gene	Forward	Reverse
narH	AAGAAGCCTGTCGAGGTTGG	CGCTTCTTGAACGCGATCTG
pflB	GCAGCATGTGAAGCGTATGG	TAGCACGTGCACCGAAGAAT
murQ	GAGAGTGCGACTGTTTCTGC	GGAATCATCGCTGGTGGACA
nagB	GTCGCAGGTACATCTTCGGT	AGAAGCAGAAGCGGAACGAT
glmS	ATACACGTTGGGCGACACAT	ACTGCAATCTCAGGTCCAGC
saeR	TGCCGGAAGTTAGCGGATAC	ATAACCAAGGCCCCACACAG

Table S2 The top fifteen upregulated differentially expressed genes (DEGs) and top fifteen downregulated DEGs of *S. epidermidis* under microoxygen condition

Gene	**Log2 fold change**	***P*adj**	***Trend***
arcC	4.212	2.16024E-25	up
argF	3.963	1.1385E-22	up
EQW00_RS01610	3.523	8.84852E-16	up
EQW00_RS01140	3.044	1.77247E-14	up
EQW00_RS07740	2.798	2.92925E-12	up
EQW00_RS05140	2.762	3.57523E-12	up
cstB	2.716	6.83081E-12	up
nrdD	2.692	1.80906E-11	up
EQW00_RS01130	2.867	1.93785E-10	up
nrdG	2.760	1.93785E-10	up
pflA	2.547	1.93785E-10	up
EQW00_RS01000	2.538	2.4346E-10	up
ureC	2.526	7.36363E-10	up
adhE	2.415	1.32353E-09	up
EQW00_RS01615	3.165	2.78954E-09	up
ppdK	-2.122	7.38881E-08	down
EQW00_RS03840	-2.808	1.72574E-07	down
vraG	-1.987	1.15305E-06	down
EQW00_RS01905	-3.185	3.24092E-06	down
EQW00_RS03820	-1.844	9.47193E-06	down
icd	-1.509	0.000265522	down
EQW00_RS03195	-1.503	0.000265522	down
EQW00_RS11405	-1.377	0.001442159	down
mraZ	-1.403	0.001454125	down
addA	-1.394	0.001458605	down
EQW00_RS06240	-1.599	0.001663391	down
rpsJ	-1.540	0.001665147	down
EQW00_RS10075	-1.481	0.001694707	down
EQW00_RS01860	-1.430	0.001695002	down
lpdA	-1.612	0.001758629	down

Table S3 The top ten upregulated differentially expressed metabolites (DEMs) and six downregulated DEMs of *S. epidermidis* under microoxygen condition

**Metabolite**	**Log2 fold change**	**VIP**	***P-Value***	***Trend***
9,10-DHOME	1.210	2.363	0.0001	up
1-(3,4-Dihydroxyphenyl)-5-hydroxy-3-decanone	0.680	2.194	0.0003	up
Lanosterin	1.240	2.175	0.0003	up
D-Lysopine	0.710	2.162	0.0004	up
(S)-Reticuline	0.570	2.078	0.0009	up
Pyroglutamic acid	0.440	2.053	0.0020	up
Procollagen 5-hydroxy-L-lysine	0.370	1.958	0.0022	up
Cis-zeatin	0.210	1.974	0.0027	up
Phosphoglycolic acid	0.300	2.098	0.0028	up
Indole-3-carboxylic acid	0.540	1.969	0.0038	up
D-Xylose	-0.660	1.880	0.0057	down
Mandelic acid	-0.350	1.695	0.0163	down
3-Dehydroshikimate	-1.120	1.766	0.0206	down
gamma-Glutamyl-gamma-aminobutyraldehyde	-1.090	1.705	0.0218	down
D-beta-Phenylalanine	-1.440	1.481	0.0438	down
6beta-Hydroxytestosterone	-0.680	1.586	0.0454	down
